# Molecular and Serologic Investigation of the 2021 COVID-19 Case Surge Among Vaccine Recipients in Mongolia

**DOI:** 10.1001/jamanetworkopen.2021.48415

**Published:** 2022-02-14

**Authors:** Naranjargal J. Dashdorj, Naranbaatar D. Dashdorj, Mitali Mishra, Lisa Danzig, Thomas Briese, W. Ian Lipkin, Nischay Mishra

**Affiliations:** 1 Onom Foundation, Ulaanbaatar, Mongolia; 2 Center for Infection and Immunity, Columbia University, New York, New York; 3 Pandefense Advisory, Mill Valley, California

## Abstract

This cross-sectional study uses molecular and serologic methods to investigate the 2021 surge in COVID-19 cases among vaccine recipients in Mongolia.

## Introduction

From February to March 2021, the number of COVID-19 cases in Mongolia (population 3.3 million) increased from less than 40 to more than 300 per day. This increase led the Mongolian government to mandate a shutdown in March 2021. A nationwide vaccination program was also initiated at that time, and 4 vaccines (2 adenovirus vectored, 1 inactivated virus, and 1 messenger RNA) were offered. This shutdown was relaxed in early June 2021 because the number of COVID-19 cases had decreased from more than 1400 per day in April to approximately 500 per day by the end of May, which was attributed to vaccination. At the end of June 2021, the number of cases increased rapidly to approximately 3000 per day (Figure).^1^

**Figure.**
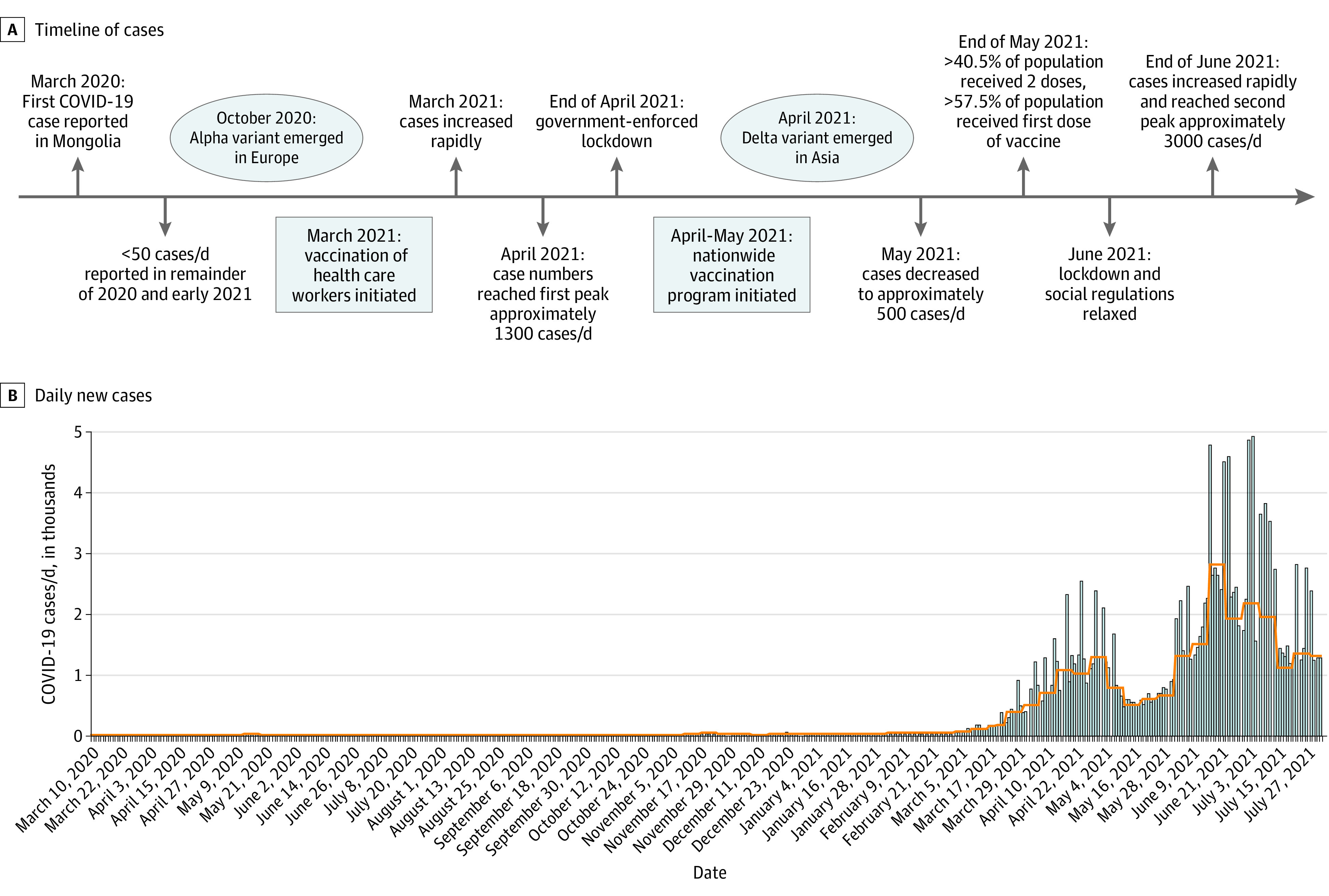
COVID-19 Cases in Mongolia, 2020 to 2021 A, Timeline of COVID-19 cases in Mongolia (February 2020 to July 2021). B, Daily new cases in Mongolia (March 2020 to July 2021) with a 7-day moving average of daily new cases (orange line). Through July 5, 2021, 2 doses of vaccine were administered to 1 800 018 individuals in Mongolia as follows: BBIBP-CotV (Sinopharm) was administered to 1 634 152 individuals, ChAdOX1-S (Covishield; Oxford/AstraZeneca) to 108 354, BNT162b2 (Pfizer/BioNTech) to 33 170, and Gam-COVID-Vac (Sputnik V; Russian Direct Investment Fund) to 24 342. Data are from Ritchie et al.^1^

This study was conducted to determine whether the surge of cases in Mongolia was associated with the emergence of the SARS-CoV-2 B.1.617.2 (Delta) variant or another SARS-CoV-2 variant or with breakthrough infections after vaccination.

## Methods

This cross-sectional study was conducted under Onom Foundation institutional review committee protocols and was approved by the Ministry of Health of Mongolia (eAppendix in the Supplement). All participants provided written informed consent. This cross-sectional study followed the Strengthening the Reporting of Observational Studies in Epidemiology (STROBE) reporting guideline.

Study participants were recruited in Mongolia between July 3 and 5, 2021. Anterior nares samples were collected from 100 hospitalized patients with a breakthrough SARS-CoV-2 infection. Plasma samples were collected from 100 healthy vaccinated individuals who did not have a prior history or any symptoms of SARS-CoV-2 infection at the time of sample collection. Illumina sequencing was performed using the myBaits capture system (Arbor Biosciences) and viral RNA from the anterior nares of patients with a breakthrough infection. Reads were mapped to the SARS-CoV-2 reference sequence (GenBank accession NC_045512), and variant analyses were performed using the GISAID database and the NextClade tool (eAppendix in the Supplement).^2^

Plasma samples were obtained from 100 healthy asymptomatic participants who had received 1 of the following 4 COVID-19 vaccines: ChAdOX1-S (adenovirus vectored, Covishield; Oxford/AstraZeneca), Gam-COVID-Vac (adenovirus vectored, Sputnik V; Russian Direct Investment Fund), BBIBP-CorV (inactivated virus; Sinopharm), or BNT162b2 (messenger RNA; Pfizer/BioNTech) (Table). The anti-SARS-CoV-2 spike-immunoglobulin G (IgG) assay and the anti-SARS-CoV-2 nucleocapsid protein (NCP)-IgG enzyme-linked immunoassay (ELISA; both from EUROIMMUN) were used to screen participant plasma for antibodies. Neutralizing antibodies to the SARS-CoV-2 Washington strain and the Delta variant were quantitated in an absolute end-point viral neutralization assay that measured 100% neutralizing activity using a SARS-CoV-2 quantitative polymerase chain reaction.^3^ Statistical analysis was performed to calculate *P* values, using a simple 2 × 2 contingency and an online χ^2^ test calculator (https://www.socscistatistics.com/tests/chisquare2/default2.aspx, version 2018, accessed November 13, 2021). The threshold for statistical significance was set at *P* < .05.

**Table.** SARS-CoV-2 Immunoreactivity of Plasma Samples From 100 Healthy Vaccinated Individuals^a^

Serologic assay result	No. of participants (%)					*P* value (total positive vs negative)^c^
	BNT162b2 (n = 24)	ChAdOX1-S (n = 24)	Gam-COVID-Vac (n = 24)	BBIBP-CorV (n = 24)	BBIBP-CorV + BNT162b2 (n = 4)^b^	
**ELISA**						
Spike						
Negative	0	5 (20.83)	12 (50.00)	17 (70.83)	0	.0002 (BNT162b2 vs Gam-COVID-Vac), .0001 (BNT162b2 vs BBIBP-CorV), .03 (ChAdOX1-S vs Gam-COVID-Vac), and .0005 (ChAdOX1-S vs BBIBP-CorV)
Total positive	24 (100.00)	19 (79.17)	12 (50.00)	7 (29.17)	4 (100.00)	
Weak positive	2 (8.33)	2 (8.33)	5 (20.83)	4 (16.67)	1 (25.00)	
Positive	22 (91.67)	17 (70.83)	7 (29.17)	3 (12.50)	3 (75.00)	
NCP						
Negative	23 (95.83)	21 (87.50)	23 (95.83)	21 (87.50)	3 (75.00)	NA
Total positive	1 (4.17)	3 (12.50)	1 (4.17)	3 (12.50)	1 (25.00)	
Weak positive	1 (4.17)	2 (8.33)	0	0	0	
Positive	0	1 (4.17)	1 (4.17)	3 (12.50)	1 (25.00)	
**Neutralizing antibody **						
Washington strain						
Not detected	5 (20.83)	12 (50.00)	18 (75.00)	19 (79.17)	1 (25.00)	.03 (BNT162b2 vs ChAdOX1-S), .0002 (BNT162b2 vs Gam-COVID-Vac), .00005 (BNT162b2 vs BBIBP-CorV), and .03 (ChAdOX1-S vs BBIBP-CorV)
Detected	19 (79.17)	12 (50.00)	6 (25.00)	5 (20.83)	3 (75.00)	
≤1:40	14 (58.30)	4 (16.70)	4 (16.70)	2 (8.33)	2 (50.00)	
>1:40	5 (20.83)	8 (33.30)	2 (8.33)	3 (12.50)	1 (25.00)	
Delta variant						
Not detected	17 (70.83)	15 (62.50)	22 (91.60)	21 (87.50)	3 (75.00)	.016 (ChAdOX1-S vs Gam-COVID-Vac) and .045 (ChAdOX1-S vs BBIBP-CorV)
Detected	7 (29.17)	9 (37.50)	2 (8.33)	3 (12.50)	1 (25.00)	
≤1:40	4 (16.67)	3 (12.50)	1 (4.20)	2 (8.33)	0	
>1:40	3 (12.50)	6 (25.00)	1 (4.20)	1 (4.20)	1 (25.00)	

^a^ Determined by the spike-immunoglobulin G enzyme-linked immunoassay (ELISA), nucleocapsid protein (NCP)-ELISA, and end point absolute viral neutralization assay against the SARS-CoV-2 Washington strain and the B.1.617.2 (Delta) variant. Participants had received 1 of 4 COVID-19 vaccines: ChAdOX1-S (Covishield; Oxford/AstraZeneca), Gam-COVID-Vac (Sputnik V; Russian Direct Investment Fund), BBIBP-CorV (Sinopharm), or BNT162b2 (Pfizer/BioNTech).

^b^ The BBIBP-CorV + BNT162b2 group was not included in the statistical analysis because of the small sample size.

^c^ *P* values were recorded for the NCP-ELISA. The χ^2^ test was used to determine significance (<.05). NA, not applicable.

## Results

In this study, we sequenced 97 anterior nares samples from hospitalized patients with a symptomatic breakthrough SARS-CoV-2 infection. Full-length SARS-CoV-2 sequences were obtained from 92 of 97 patient samples with sufficient RNA. Of the 92 sequences, we identified 87 (94.60%) as the B.1.1.7 (Alpha) variant and 5 (5.40%) as the Delta variant. Of the 87 patients with an Alpha variant breakthrough infection, 54 (62.10%) had received the BBIBP-CorV vaccine, 23 (26.40%) the ChAdOX1-S vaccine, 7 (8.00%) the Gam-COVID-Vac vaccine, and 3 (3.40%) the BNT162b2 vaccine. Of the 5 patients with a breakthrough Delta variant infection, 1 (20.00%) had received the BBIBP-CorV vaccine, 3 (60.00%) the ChAdOX1-S vaccine, and 1 (20.00%) the BNT162b2 vaccine. At the time of sample collection, more than 1.8 million individuals (90.00%) had received the BBIBP-CorV vaccine.

Of the 100 healthy asymptomatic participants, more individuals had a reactive spike-ELISA result after they received the BNT162b2 (24 [100.00%]) or ChAdOX1-S (19 [79.17%]) vaccine compared with those who received the Gam-COVID-Vac (12 [50.00%]) or BBIBP-CorV (7 [29.17%]) vaccine. Three BBIBP-CorV vaccine recipients (12.50%) had a reactive NCP-ELISA result. Three ChAdOX1-S vaccine recipients (12.50%) had a positive NCP result; this suggested a past SARS-CoV-2 infection, because currently the ChAdOX1-S vaccine contains the only spike protein. Only BBIBP-CorV, an inactivated whole virus vaccine, contains NCP. Of 4 participants who had received 2 BBIBP-CorV doses followed by 1 BNT162b2 dose, 1 (25.00%) had a weak spike-ELISA result (Table).

More participants had 100% end-point neutralizing antibodies to the Washington strain after they received the BNT162b2 (19 [79.17%]) or ChAdOX1-S (12 [50.00%]) vaccine compared with those who received the Gam-COVID-Vac (6 [25.00%]) or BBIBP-CorV (5 [20.83%]) vaccine. In addition, 100% end-point neutralizing antibodies to the Delta variant were present in fewer participants after they received the BNT162b2 (7 [29.17%]), Gam-COVID-Vac (2 [8.33%]), or BBIBP-CorV (3 [12.5%]) vaccine. Nine recipients (37.50%) had 100% end-point neutralizing antibodies to the Delta variant after they received the ChAdOX1-S vaccine. However, 3 of 9 participants (12.50%) had a positive NCP-ELISA result, indicating a previous SARS-CoV-2 exposure. Eliminating these 3 individuals would decrease the percentage of participants with 100% end-point neutralizing antibodies to the Delta variant to 25.00%. Of 4 individuals who received a single dose of BNT162b2 vaccine after 2 doses of BBIBP-CorV vaccine, 3 (75.00%) had 100% end-point neutralizing antibodies to the Washington strain and 1 (25.00%) had neutralizing antibodies to the Delta variant (Table).

## Discussion

This cross-sectional study was limited in terms of the number of participants and its retrospective collection of specimens. Nonetheless, our findings provide support for previously published literature suggesting a hierarchy of protection among vaccine platforms, with messenger RNA vaccines performing better than adenovirus-vectored or inactivated-virus vaccines against SARS-CoV-2 infection.^4^ Our data also suggest that the current COVID-19 vaccines, based on the prototypic Washington strain, elicit only minimal levels of 100% end-point neutralizing antibodies to currently circulating strains.^5,6^

Our results suggest that the surge in COVID-19 cases in Mongolia in June 2021 was not associated with the introduction of the Delta variant but was more likely attributable to relaxed physical distancing and masking requirements. These findings underscore the importance of continuous surveillance for virus evolution, coordinated modification of vaccines, and a cautious approach to the elimination of classic social controls for mitigating contagion.

## Notes

Supplement.
eAppendix.

eReferences
