# Supplementary material for: Molecular and Serologic Investigation of the 2021 COVID-19 Case Surge Among Vaccine Recipients in Mongolia
Source: JAMA Netw Open. 2022 Feb 14;5(2):e2148415. doi: 10.1001/jamanetworkopen.2021.48415 (PMC8845004; doi:10.1001/jamanetworkopen.2021.48415)
Supplement: Supplement. — eAppendix. eReferences [file jamanetwopen-e2148415-s001.pdf]

Supplementary Online Content

Dashdorj NJ, Dashdorj ND, Mishra M, et al. Molecular and serologic investigation of the 2021 COVID-19 case surge among vaccine recipients in Mongolia. *JAMA Netw Open*. 2022;5(2):e2148415. doi:10.1001/jamanetworkopen.2021.48415

eAppendix.
eReferences

This supplementary material has been provided by the authors to give readers additional information about their work.

© 2022 Dashdorj NJ et al. *JAMA Network Open*.

eAppendix

Samples

Anterior nares samples from subjects with breakthrough infections (n=97)

Samples were collected with written informed consent under the auspices of Onom IRB # 241 and IRB # 242. We obtained anterior nares swabs from 97 subjects with breakthrough infections after vaccination. Fifty-two (53.6%) patients females (average age: 42.8±13.8 years); 45 (48.4%) were males (average age: 41.9±15.5 years). Eighty-four (86.6%) were fully vaccinated i.e. received both (prime and booster) doses of vaccines; 13 (13.4%) received only the first (prime) dose of vaccine prior to breakthrough infection. Out of 84 who received both doses, 53 (63.1%) received BBIBP-CorV, 28 (33.3%) received ChAdOX1-S, 2 (2.4%) received Gam-COVID-Vac, and one (1.2%) received BNT162b2.

Plasma samples from healthy vaccinated individuals (n=100)

Samples were collected with written informed consent under the auspices of Onom IRB # 241 and IRB # 242. Of 100 subjects, 24 (24%) received two doses of BBIBP-CorV, 24 (24%) received two doses of ChAdOX1-S, 24 (24%) received two doses of Gam-COVID-Vac, 24 (24%) two doses of BNT162b2 (prime and booster); 4 (4%) received two doses of BBIBP-CorV and one dose of BNT162b2. All samples were tested for anti-spike SARS-CoV-2 antibodies, and anti-nucleocapsid-phosphoprotein SARS-CoV-2 antibodies using EUROIMMUN ELISA kits, and for neutralizing antibodies using an end-point viral neutralization assay [1].

© 2022 Dashdorj NJ et al. *JAMA Network Open*.

SARS-CoV-2 Sequencing

RNA extracted using QIAcube HT (Qiagen) was employed for SARS-CoV-2 complete genomic sequencing. Illumina libraries were prepared and enriched for SARS-CoV-2 sequences using Mybaits capture system (Arbor Biosciences) [2]. Captured libraries were pooled and sequenced on the Illumina Nextseq 2000; and ~6-8 million reads/samples were generated. After demultiplexing, reads were mapped and aligned against the SARS-CoV-2 reference sequence (accession no. NC_045512).

© 2022 Dashdorj NJ et al. *JAMA Network Open*.

eReferences

1. Eckhardt CM, Cummings MJ, Rajagopalan KN, et al. Evaluating the efficacy and safety of human anti-SARS-CoV-2 convalescent plasma in severely ill adults with COVID-19: A structured summary of a study protocol for a randomized controlled trial. Trials 2020;**21**(1):499 doi: 10.1186/s13063-020-04422-y[published Online First: Epub Date]|.

2. Tillett RL, Sevinsky JR, Hartley PD, et al. Genomic evidence for reinfection with SARS-CoV-2: a case study. Lancet Infect Dis 2021;**21**(1):52-58 doi: 10.1016/S1473-3099(20)30764-7[published Online First: Epub Date]|.

© 2022 Dashdorj NJ et al. *JAMA Network Open*.
